# Supplementary figures and images for: Misreporting contraceptive use and the association of peak study progestin levels with weight and BMI among women randomized to the progestin-only injectable contraceptives DMPA-IM and NET-EN
Source: PLoS One. 2023 Dec 22;18(12):e0295959. doi: 10.1371/journal.pone.0295959 (PMC10745193; doi:10.1371/journal.pone.0295959)

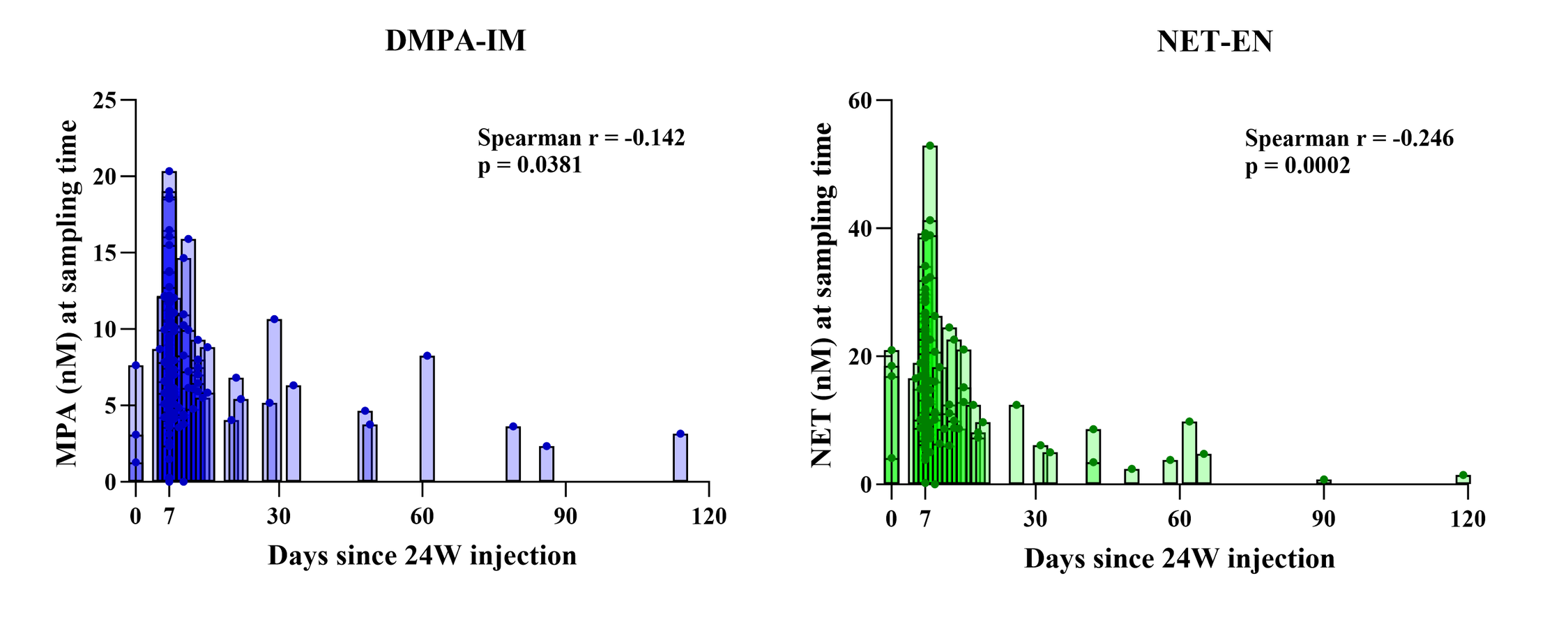

Supplement: S1 Fig — Graphs indicate days since the last DMPA-IM (A) or NET-EN (B) injection at 24 weeks (24W) and when blood was collected for determination of serum MPA or NET concentrations plotted against the determined serum MPA (nM) (A) or NET (nM) (B) concentrations in the DMPA-IM or NET-EN arms, respectively, for each participant. Non-parametric Spearman correlation analysis was performed and Spearman r- and p-values for the correlations are given. (TIF) [file pone.0295959.s001.tif]
