# Supplementary material for: Misreporting contraceptive use and the association of peak study progestin levels with weight and BMI among women randomized to the progestin-only injectable contraceptives DMPA-IM and NET-EN
Source: PLoS One. 2023 Dec 22;18(12):e0295959. doi: 10.1371/journal.pone.0295959 (PMC10745193; doi:10.1371/journal.pone.0295959)
Supplement: S1 Table — (DOCX) [file pone.0295959.s002.docx]

**S1 Table. Nomenclature, retention times, quantifier and qualifier mass transitions, collision energies and cone voltages of target analytes and internal standards.**

|  |  | **Retention time**  **(min)** | **Quantifier**  ***Qualifier***  **(m/z)** | **Cone voltage**  **(V)** | **Collision energy**  **(eV)** |
| --- | --- | --- | --- | --- | --- |
| **MPA** | Medroxyprogesterone acetate | 3.81 | 387.2 > 123.1 | 30 | 20 |
|  |  |  | *387.2 > 285.15* | 30 | 20 |
| **NET** | Norethisterone | 2.31 | 299 > 109 | 30 | 30 |
|  |  |  | *299 > 231* | 30 | 20 |
| **LNG** | Levonorgestrel | 2.97 | 313 > 109 | 20 | 25 |
|  |  |  | *313 > 245* | 25 | 30 |
| **ETG** | Etonogestrel | 3.19 | 325.2 > 109 | 25 | 30 |
|  |  |  | *325.2 > 147* | 25 | 25 |
| **NES** | Nestorone | 2.98 | 371.2 > 253.2 | 25 | 20 |
|  |  |  | *371.2 > 269* | 25 | 25 |
| **GES** | Gestodene | 2.41 | 311.2 > 135 | 15 | 25 |
|  |  |  | *311.2 > 109.4* | 15 | 25 |
| **MPA-d6^*^** | Medroxyprogesterone-6,6,6-d3 17-acetate-2,2,2-d3 | 3.78 | 393.1 > 288.15 | 25 | 30 |
|  |  |  | *393.1 > 330.2* | 25 | 30 |
| **NET-d6^*^** | Norethindrone-2,2,4,6,6,10-d6 | 2.28 | 305.05 > 113.2 | 25 | 30 |
|  |  |  | *305.05 > 237.25* | 25 | 30 |

^*^d = deuterium
