# Supplementary material for: Misreporting contraceptive use and the association of peak study progestin levels with weight and BMI among women randomized to the progestin-only injectable contraceptives DMPA-IM and NET-EN
Source: PLoS One. 2023 Dec 22;18(12):e0295959. doi: 10.1371/journal.pone.0295959 (PMC10745193; doi:10.1371/journal.pone.0295959)
Supplement: S2 Table — (DOCX) [file pone.0295959.s003.docx]

**S2 Table. Comprehensive method validation data^*^.**

|  | **R^2^** | **Accuracy**  **(% BIAS)** | | | | **Precision intra assay**  **(% CV)** | | | | **Precision inter assay**  **(% CV)** | | | | **Matrix effect (%)** | **Extraction efficiency (%)** |
| --- | --- | --- | --- | --- | --- | --- | --- | --- | --- | --- | --- | --- | --- | --- | --- |
|  |  | **0.05 ng/mL** | **0.1 ng/mL** | **0.5 ng/mL** | **50 ng/mL** | **0.05 ng/mL** | **0.1 ng/mL** | **0.5 ng/mL** | **50 ng/mL** | **0.05 ng/mL** | **0.5 ng/mL** | **5 ng/mL** | **50 ng/mL** | **5 ng/mL** | **5 ng/mL** |
| **MPA** | 0.996 | 3.78 | 4.00 | -3.06 | 5.97 | 9.88 | 1.73 | 6.59 | 3.25 | 12.8 | 7.66 | 5.56 | 3.68 | 5.44 | 84.9 |
| **NET** | 0.997 | 9.75 | 0.13 | -2.28 | 10.0 | 1.38 | 7.72 | 5.82 | 2.06 | 13.9 | 6.66 | 7.90 | 4.17 | 4.78 | 85.1 |
| **LNG** | 0.998 | - | -7.62 | -6.10 | -2.20 | - | 10.8 | 5.84 | 1.34 | - | 13.3 | 5.12 | 4.68 | 10.8 | 81.7 |
| **ETG** | 0.996 | - | 10.0 | -4.70 | 0.40 | - | 3.57 | 1.14 | 4.60 | - | 9.50 | 3.39 | 3.35 | 7.70 | 82.8 |
| **NES** | 0.998 | 8.75 | 8.40 | -7.56 | -4.17 | 14.5 | 9.84 | 3.76 | 2.09 | 11.4 | 7.07 | 4.66 | 3.86 | 15.1 | 84.1 |
| **GES** | 0.998 | - | - | -0.68 | -8.68 | - | - | 2.53 | 0.98 | - | 10.9 | 9.47 | 5.59 | -4.13 | 92.0 |

^*^R^2^, accuracy, precision, matrix effects and extraction efficiency are shown for stripped human serum. (-) indicates that the concentration is below the LLOQ for that steroid and is therefore not included. All n = 10. CV: coefficient of variation.
