# Supplementary material for: Misreporting contraceptive use and the association of peak study progestin levels with weight and BMI among women randomized to the progestin-only injectable contraceptives DMPA-IM and NET-EN
Source: PLoS One. 2023 Dec 22;18(12):e0295959. doi: 10.1371/journal.pone.0295959 (PMC10745193; doi:10.1371/journal.pone.0295959)
Supplement: S3 Table — (DOCX) [file pone.0295959.s004.docx]

**S3 Table.** **Limit of Detection (LOD), lower and upper limit of quantification (LLOQ and ULOQ, respectively)^*^.**

|  | **LOD** | | **LLOQ** | | **ULOQ** | |
| --- | --- | --- | --- | --- | --- | --- |
|  | **ng/mL** | **nM** | **ng/mL** | **nM** | **ng/mL** | **nM** |
| **MPA** | 0.0100 | 0.0259 | 0.0500 | 0.129 | 50.0 | 129 |
| **NET** | 0.0250 | 0.0838 | 0.0500 | 0.168 | 50.0 | 168 |
| **LNG** | 0.0100 | 0.0320 | 0.100 | 0.320 | 50.0 | 160 |
| **ETG** | 0.0500 | 0.154 | 0.100 | 0.308 | 50.0 | 154 |
| **NES** | 0.0250 | 0.0675 | 0.0500 | 0.135 | 50.0 | 135 |
| **GES** | 0.500 | 1.611 | 0.500 | 1.61 | 50.0 | 161 |

^*^LOD (ng/mL, nM), LLOQ (ng/mL, nM) and ULOQ (ng/mL, nM) were determined as described in the methods.
