# Supplementary material for: Misreporting contraceptive use and the association of peak study progestin levels with weight and BMI among women randomized to the progestin-only injectable contraceptives DMPA-IM and NET-EN
Source: PLoS One. 2023 Dec 22;18(12):e0295959. doi: 10.1371/journal.pone.0295959 (PMC10745193; doi:10.1371/journal.pone.0295959)
Supplement: S4 Table — (DOCX) [file pone.0295959.s005.docx]

**S4 Table. Mean and range of imputed MPA and NET concentrations for whole cohort (mITT).**

|  | **DMPA-IM** | | **NET-EN** | | **DMPA-IM vs NET-EN** |
| --- | --- | --- | --- | --- | --- |
|  | **Mean (Min; Max)** | **n** | **Mean (Min; Max)** | **n** | **Site-adjusted p-value^#^** |
| **MPA (nM)** | | | | | |
| D0 | 1.16 (0.00; 14.3) | 215 | 1.32 (0.00; 13.1) | 220 | 0.355 |
| 25W | 7.03 (0.00; 20.4) | 215 | 0.278 (0.00; 20.3) | 220 | **<0.001** |
| Change (25W - D0) | 5.90 (-7.11; 19.4) |  | -1.04 (-12.5; 20.1) |  | **<0.001** |
| Change site-adjusted p-value# | **<0.001** | | **<0.001** | |  |
| **NET (nM)** | | | | | |
| D0 | 0.348 (0.00; 7.51) | 215 | 0.248 (0.00; 16.9) | 220 | 0.066 |
| 25W | 0.329 (0.00; 20.7) | 215 | 14.9 (0.00; 52.9) | 220 | **<0.001** |
| Change (25W - D0) | -0.0209 (-3.09; 20.7) |  | 14.6 (-16.7; 52.7) |  | **<0.001** |
| Change site-adjusted p-value^#^ | **<0.001** | | **<0.001** | |  |

^#^Site-adjusted p-values were obtained by generalized linear models using the Box-Cox power transformation of progestin data.
