# Supplementary material for: Misreporting contraceptive use and the association of peak study progestin levels with weight and BMI among women randomized to the progestin-only injectable contraceptives DMPA-IM and NET-EN
Source: PLoS One. 2023 Dec 22;18(12):e0295959. doi: 10.1371/journal.pone.0295959 (PMC10745193; doi:10.1371/journal.pone.0295959)
Supplement: S5 Table — (DOCX) [file pone.0295959.s006.docx]

**S5 Table. Time (days) between final injection (24W) and sampling time.**

|  | **DMPA-IM** | | | **NET-EN** | | | **DMPA-IM vs NET-EN** |
| --- | --- | --- | --- | --- | --- | --- | --- |
|  | **Median (IQR)** | **Mean**  **(Min; Max)** | **n** | **Median (IQR)** | **Mean**  **(Min; Max)** | **n** | **p-value^*^** |
| **Sampling time (days) since 24W injection** | 7.00  (7.00; 8.00) | 13.4  (0.00; 188) | 217 | 7.00  (7.00; 7.0) | 11.3  (0.00; 225) | 219 | 0.743 |

^*^Unadjusted; Between group analysis - Mann Whitney test.
