# Supplementary material for: Misreporting contraceptive use and the association of peak study progestin levels with weight and BMI among women randomized to the progestin-only injectable contraceptives DMPA-IM and NET-EN
Source: PLoS One. 2023 Dec 22;18(12):e0295959. doi: 10.1371/journal.pone.0295959 (PMC10745193; doi:10.1371/journal.pone.0295959)
Supplement: S6 Table — (DOCX) [file pone.0295959.s007.docx]

**S6 Table. Non-study contraceptive use per study site for the whole cohort (mITT).**

|  | **MRU** | | | **ECRU** | | |
| --- | --- | --- | --- | --- | --- | --- |
|  | **(n = 150)** | | | **(n = 285)** | | |
|  | **n*** | **%^#^** | **Median (IQR) nM** | **n*** | **%^#^** | **Median (IQR) nM** |
| **MPA** | | | | | | |
| D0 | 78 | 52.0 | 1.18 (0.434;3.44) | 157 | 55.1 | 1.32 (0.490; 2.79) |
| 25W^$^ | 22 | 30.1 | 0.334 (0.204; 0.996) | 37 | 25.3 | 0.461 (0.311; 0.787) |
| **NET** | | | | | | |
| D0 | 42 | 28.0 | 0.401 (0.277; 1.39) | 82 | 28.8 | 0.416 (0.286; 1.03) |
| 25W^$$^ | 16 | 20.8 | 0.530 (0.303; 3.76) | 24 | 17.3 | 0.396 (0.277; 0.857) |
| **LNG** | | | | | | |
| D0 | 14 | 9.33 | 1.51 (1.12; 1.89) | 16 | 5.61 | 0.736 (0.509; 2.40) |
| 25W | 6 | 4.00 | 2.34 (0.820; 6.19) | 9 | 3.16 | 0.602 (0.437; 1.35) |
| **ETG** | | | | | | |
| D0 | 3 | 2.00 | 1.37 (0.712; 1.84) | 2 | 0.702 | 0.565 (0.508; 0.622) |
| 25W | 2 | 1.33 | 0.673 (0.545; 0.801) | 1 | 0.3510 | 1.02 (1.02; 1.02) |
| **NES** | | | | | | |
| D0 | 3 | 2.00 | 0.229 (0.140; 2.08) | 5 | 1.75 | 0.291 (0.204; 0.520) |
| 25W | 6 | 4.00 | 0.310 (0.174; 0.389) | 8 | 2.81 | 0.216 (0.173; 0.864) |
| **GES** | | | | | | |
| D0 | 1 | 0.667 | 1.77 (1.77; 1.77) | 1 | 0.351 | 2.54 (2.54; 2.54) |
| 25W | 0 | 0 | 0.00 (0.00; 0.00) | 2 | 0.702 | 4.40 (3.84; 4.96) |


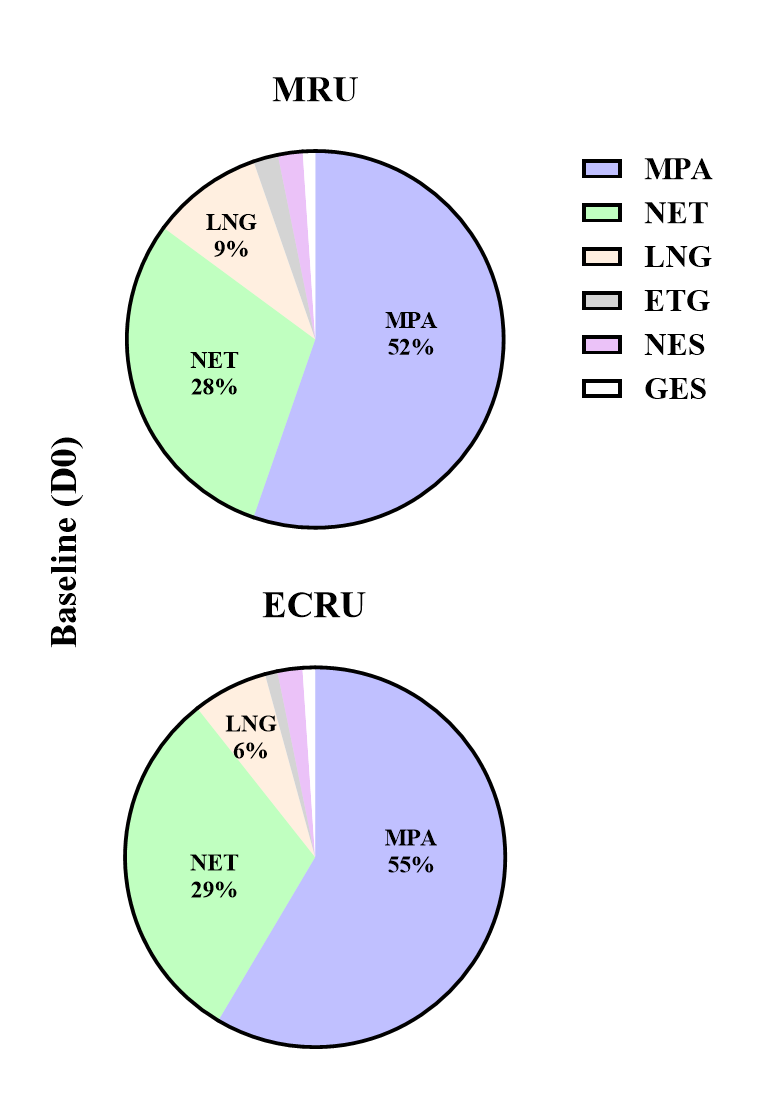


*n-values given are the number of participants that had specified progestin at concentrations > LLOQ at either the MRU (n = 150 total) or the ECRU site (n = 285 total). **^#^** % refers to the percentage of participants that had specified progestin at concentrations > LLOQ in the respective site. ^$^In NET-EN arm only (total MRU = 73 total ECRU = 146); ^$$^In DMPA-IM arm only (total MRU participants = 77, total ECRU participants = 139).
