# Supplementary material for: Misreporting contraceptive use and the association of peak study progestin levels with weight and BMI among women randomized to the progestin-only injectable contraceptives DMPA-IM and NET-EN
Source: PLoS One. 2023 Dec 22;18(12):e0295959. doi: 10.1371/journal.pone.0295959 (PMC10745193; doi:10.1371/journal.pone.0295959)
Supplement: S7 Table — (DOCX) [file pone.0295959.s008.docx]

**S7 Table. Mean and range of imputed MPA and NET concentrations analyzed for subgroup of the whole cohort (PP1).**

|  | **DMPA-IM** | | **NET-EN** | | **DMPA-IM vs NET-EN** |
| --- | --- | --- | --- | --- | --- |
|  | **Mean (Min; Max)** | **n** | **Mean (Min; Max)** | **n** | **Site-adjusted p-value^#^** |
| **MPA (nM)** | | | | | |
| D0 | 1.178 (0.00; 14.3) | 161 | 0.772 (0.00; 11.7) | 155 | **0.003** |
| 25W | 7.46 (0.168; 20.4) | 161 | 0.0191 (0.00; 0.0650) | 155 | **<0.001** |
| Change (25W - D0) | 6.28 (-7.11; 20.1) |  | -0.753 (-11.7; 0.0650) |  | **<0.001** |
| Change site-adjusted p-value^#^ | **<0.001** | | **<0.001** | |  |
| **NET (nM)** | | | | | |
| D0 | 0.155 (0.00; 3.09) | 161 | 0.155 (0.00; 3.85) | 155 | 0.976 |
| 25W | 0.0121 (0.00; 0.111) | 161 | 14.8 (2.42; 52.9) | 155 | **<0.001** |
| Change (25W - D0) | -0.143 (-3.00; 0.0840) |  | 14.6 (2.42; 52.7) |  | **<0.001** |
| Change site-adjusted p-value^#^ | **<0.001** | | **<0.001** | |  |

^#^Site-adjusted p-values were obtained by generalized linear models using the Box-Cox power transformation for progestin data.
