# Supplementary material for: Misreporting contraceptive use and the association of peak study progestin levels with weight and BMI among women randomized to the progestin-only injectable contraceptives DMPA-IM and NET-EN
Source: PLoS One. 2023 Dec 22;18(12):e0295959. doi: 10.1371/journal.pone.0295959 (PMC10745193; doi:10.1371/journal.pone.0295959)
Supplement: S8 Table — (DOCX) [file pone.0295959.s009.docx]

**S8 Table. Time varying associations between the change (25W-D0) in MPA or NET concentrations with change in baseline weight or BMI.**

|  | **mITT** | | **PP2** | |
| --- | --- | --- | --- | --- |
|  | **MPA*** | **NET#** | **MPA*** | **NET#** |
|  | **Change (25W-D0)** | **Change (25W-D0)** | **Change (25W-D0)** | **Change (25W-D0)** |
| **D0 Weight (kg)** | **0.021 ↓** | **<0.001 ↓** | **0.010 ↓** | **<0.001 ↓** |
| **D0 BMI** | **0.010 ↓** | **<0.001 ↓** | **0.005 ↓** | **<0.001 ↓** |

*In the DMPA-IM arm only; ^#^In the NET-EN arm only: P-values for time-varying associations were obtained by generalized linear models using Box-Cox power transformations for the progestin data.
