# Supplementary material for: Misreporting contraceptive use and the association of peak study progestin levels with weight and BMI among women randomized to the progestin-only injectable contraceptives DMPA-IM and NET-EN
Source: PLoS One. 2023 Dec 22;18(12):e0295959. doi: 10.1371/journal.pone.0295959 (PMC10745193; doi:10.1371/journal.pone.0295959)
Supplement: S9 Table — (DOCX) [file pone.0295959.s010.docx]

**S9 Table. Estimated percentage of women misreporting DMPA-IM or NET-EN use or possible ‘tail’ effects.**

|  | **MPA** | | | **NET** | | |
| --- | --- | --- | --- | --- | --- | --- |
|  | **Total** | **Tail** | **Misreport / active use*** | **Total** | **Tail** | **Misreport / active use*** |
| **D0 Whole cohort^#^ n = 435** | n = 236 (54.3%) | n = 125 (28.7%) | n = 111 (25.5%) | n = 124 (28.5%) | n = 95 (21.8%) | n = 29 (6.67%) |
| **25W DMPA-IM arm only^$^ n = 215** | NA | | | n = 40 (18.6%) | n = 18 (8.37%) | n = 22 (10.2%) |
| **25W NET-EN arm only^$$^ n = 220** | n = 60 (27.3%) | n = 18 (8.18%) | n = 42 (19.1%) | NA | | |

^#^ For MPA detected at D0: Based on modelling [1], a ‘tail’ resulting from one DMPA-IM injection six months before initiation would result in a concentration of about 0.26 – 1.4 nM at D0. We thus conservatively estimated that ‘tail’ effects were the likely cause of participants at D0 with quantifiable MPA < 1.4 nM for the whole cohort. We conservatively estimated that participants with MPA concentrations > 1.4 nM at D0, most likely misreported receiving no DMPA-IM injection less than six months before initiation.

^#^ For NET detected at D0, based on limited information in the literature [2], we estimated that women with NET > 1.17 nM at D0 were likely to have misreported no NET-EN use four months prior to initiation. The reason for women with NET concentrations < 1.17 nM at D0 we therefore estimated as being likely due to NET-EN ‘tail’ effects.

^$^ For MPA detected at 25W in the NET-EN arm, based on modelling [1], we estimated that a ‘tail’ resulting from one DMPA-IM injection six months before initiation would result in a concentration of < 0.26 nM. We estimated that for MPA concentrations > 0.26 nM at 25W in the NET-EN arm, women either misreported receiving one DMPA-IM injection less than six months before initiation (therefore having a hormone ‘tail’ due to misreporting) or received at least one DMPA-IM injection after D0, which would be misreporting active use of non-study progestin during the trial.

^$$^ For NET detected at 25W in the DMPA-IM arm, based on limited information [2], we estimated that a ‘tail’ resulting from one NET-EN injection four months before initiation would result in a concentration of ≤ 0.37 nM. We estimated that for NET concentrations > 0.37 nM at 25W in the DMPA-IM arm, women either misreported receiving one NET-EN injection less than four months before initiation (therefore having a NET ‘tail’ due to misreporting) or received at least one NET-EN injection after D0, which would be misreporting active use of non-study progestin during the trial.

*When looking at D0 (initiation) the n- (%) values indicate misreporting of contraceptive use during the four – six months before trial initiation, while for 25W samples, the n- (%) values are a combination of active contraceptive use during the trial and potential hormone ‘tails’ from misreporting prior to initiation.

These values are estimates that should be interpreted with caution given the limited pharmacokinetic data available for both contraceptives. It is also possible that at least some participants exhibited atypical pharmacokinetic profiles.

**References**

1. Evidence for Contraceptive Options and HIV Outcomes (ECHO) Trial Consortium. HIV incidence among women using intramuscular depot medroxyprogesterone acetate, a copper intrauterine device, or a levonorgestrel implant for contraception: a randomised, multicentre, open-label trial. Lancet. 2019; 394(10195):303-13.

2. Goebelsmann U, Stanczyk FZ, Brenner PF, Goebelsmann AE, Gentzschein EK, Mishell DR, Jr. Serum norethindrone (NET) concentrations following intramuscular NET enanthate injection. Effect upon serum LH, FSH, estradiol and progesterone. Contraception. 1979; 19(3):283-313.
