# Supplementary material for: Misreporting contraceptive use and the association of peak study progestin levels with weight and BMI among women randomized to the progestin-only injectable contraceptives DMPA-IM and NET-EN
Source: PLoS One. 2023 Dec 22;18(12):e0295959. doi: 10.1371/journal.pone.0295959 (PMC10745193; doi:10.1371/journal.pone.0295959)
Supplement: S1 Protocol — (DOCX) [file pone.0295959.s011.docx]

**S1 Protocol**

**WHICH clinical trial additional methods**

**Aim, design and setting**

We aimed to investigate the effects of DMPA-IM and NET-EN on estradiol levels and menstrual, psychological and behavioral measures relevant to HIV risk. Towards this goal we conducted a parallel, open label, individually-randomized trial at the East London and Mdantsane public health clinics and hospitals (Frere and Cecilia Makiwane Hospitals), South Africa (331 participants), and the research site of MatCH Research Unit (MRU), University of the Witwatersrand, based in Durban, KwaZulu-Natal, South Africa (189 participants). A summarized protocol is available at <https://pactr.samrc.ac.za/TrialDisplay.aspx?TrialID=6073>.

**Participants**

We recruited participants attending family planning clinics and those in the local communities who requested injectable contraception and intended to continue contraception for at least 18 months; were aged 18 to 40 years; legally competent to sign consent according to local regulations; prepared to use either DMPA-IM or NET-EN; prepared to accept follow-up procedures and able to fulfil these procedures, including routine HIV tests according to national guidelines; who after full counselling declined to use pre-exposure prophylaxis (PrEP) for HIV; understood the patient information form and signed written informed consent. Exclusion criteria were participants who had received DMPA-IM in the previous 6 months or NET-EN in the previous 4 months; were HIV positive; were planning to move out of the study area in the next 18 months; were participating in another clinical trial; were <6 weeks postpartum or post-abortion; had diabetes or high blood pressure; did not meet the WHO medical eligibility criteria (MEC) or local national guidelines for DMPA-IM or NET-EN use; or were using or intending to use medication which might have interfered with biological measurements such as steroids or drugs affecting renal function such as PrEP. Prospective participants were fully informed about PrEP, and if interested in using PrEP, were referred to a local provider. Participants were recruited and followed from 5 November 2018 to 30 November 2019. Participants who, after recruitment, changed their minds and decided to access PrEP services, were to remain in the study. To our knowledge, none did. Participants who met the entry criteria were fully counselled and informed in their preferred language and invited to participate. Participants were counselled on HIV risk reduction including condom use.

Exclusion of pregnancy and clinical assessment for sexually transmitted infections or contra-indications to the contraceptives were conducted, and any illness or pregnancy detected was managed in the routine service.

**Randomisation and masking**

Allocation lists were prepared independently by SA Medical Research Council (MRC) using computer-generated random sequence in balanced blocks of variable size, stratified by study site. Participants who agreed to participate were entered onto a trial register and then randomized by accessing the online randomization REDCap programme [1]. In the event of difficulty accessing the online service, a separate series of randomized allocations was available in sequentially numbered, sealed opaque envelopes, or by telephone back-up service. Participants and research staff administering treatments were not masked to group allocation. Those conducting outcome interviews were not aware of the group allocation of participants, but this could have become apparent during some interviews.

**Procedures**

Baseline demographic, menstrual, psychological, and behavioral data were recorded before randomization. Each participant was assigned a unique participant trial identification number (PTID) and data were collected using the PTID. Authors did not have access to information that could identify individual participants during or after data collection. Baseline blood (up to 40 ml venous blood), dried blood spots and genital tract samples (cervical cytobrush and lateral vaginal wall swabs) for ancillary future immunological and hormonal studies and archiving were collected. Blood samples were separated and the serum stored at -80°C. Participants were allocated to receive DMPA-IM 150 mg intramuscular 12-weekly or NET-EN 200 mg intramuscular 8-weekly. Strategies to manage side-effects without method change were explored with participants. In the event of discontinuation of either method, alternative choices were offered to participants according to national contraception guidelines. Participants were asked to attend the research sites at the time of their repeat injections (8- or 12-weekly) to 24 weeks, and at 25 weeks to collect 7-day post-injection biological samples. A 28-day daily symptom and behavior diary was initiated at 24 weeks. At the final study visit, the participants were re-counselled about their future contraceptive choices. Further contraceptive care was provided within the routine provincial health services. Biological samples were collected and questionnaires administered at 25 weeks and participants were offered an HIV test by study staff, in line with national guidelines. Participants received approved compensation for their time and costs for in-person visits (R250 for study visits and R100 for contraception-only provision visits). Every attempt was made to contact participants who did not return for follow up including repeated calls to participant’s and alternative phone numbers, and where possible home visits (provided previously consented to). Participants who acquired HIV were referred for HIV care to local healthcare facilities. Those who had depressive symptoms were counselled and referred.

**Outcomes**

The primary laboratory outcome was serum 17β estradiol, and the primary clinical outcome was depression score (Beck Depression Inventory - BDI-II).

Estradiol was measured at Neuberg Global Laboratories (Durban, KwaZulu Natal, South Africa) by a chemiluminescent microparticle immunoassay (ARCHITECT Estradiol B7K720, analytical sensitivity ≤ 10 pg/mL) on stored baseline and 25-week (7 days after the 24-week injection) serum samples. HIV assays (finger prick, rapid HIV test) were performed on site. Additional hormonal and immunological studies will be reported separately. The BDI-II method was chosen to evaluate depressive symptoms. It has previously been validated and used in the same cultural context and translated into the local languages IsiXhosa and IsiZulu. English, IsiZulu and IsiXhosa versions were used. Verbal administration was utilised. The BDI-II has 21 items, and each item is rated on a four-point scale ranging from 0-3. The maximum total score is 63. According to the BDI-II manual, scores of 0-13 indicate no or minimal depression, scores of 14-19 indicate mild depression, scores of 20-28 indicate moderate depression, and scores of 29-63 indicate severe depression [2]. The Arizona Sexual Experiences Scale (ASEX) was used to evaluate sexual function. This is a five-item rating scale with total scores ranging from 5–30. It has been validated to be independent of the presence of a coital partner and can therefore be used even when study participants are not coitally active. Questions 4 and 5 of this scale are not ranked if a participant has not engaged in sexual intercourse within a week of the interview. A structured questionnaire was used to assess other secondary psychological and behavioral parameters: feeling sad for no reason, no menstruation, painless menstruation, no sexual intercourse, never use a condom during intercourse, and decreased sexual desire. Participants were asked to prospectively complete a 28-day daily diary at home of symptoms and behavior, commencing on the day of their 24-week visit. Parameters measured in the daily diary were: characteristics of menstruation, sexual intercourse with steady or casual partner, condom use, feeling sad for no reason, feeling the urge to have sexual intercourse, and feeling that partner loves her. The trial was not powered for HIV acquisition or pregnancy, but these were measured to provide an incidence estimate to inform a potential future larger trial.

**Statistical analysis**

The primary laboratory outcome was serum estradiol. In a previous study [3], estradiol levels in postpartum participants randomized to NET-EN were 136 pmol/L (standard deviation (SD) 119). Based on this SD, to show a difference of 35 pmol/L in either direction between groups with 95% certainty and 80% power would require 181 participants per group, and allowing for 15% loss to follow-up, 213 per group (<http://pharmaschool.co/size4.asp>).

The primary clinical outcome was depression score. In a previous study [4], the Montgomery-Asberg Depression Rating Scale (MADRS) scores in postpartum participants randomized to NET-EN were 8.3 (standard deviation (SD) 7.5). Based on this SD, to show a difference of 2 between the study groups with 95% certainty and 80% power would require 221 participants per group, and allowing for 15% loss to follow-up, 260 per group. (<http://pharmaschool.co/size4.asp>).

All measured clinical outcomes were reported, and secondary outcome comparisons were regarded as exploratory.

Statistical analysis was by intention to treat (ITT), and the results are reported according to the CONSORT guidelines. Data were analysed using Stata 16 (StataCorp, College Station, TX, USA). Descriptive statistics are presented as frequencies with percentages and means with standard deviations (SD). Where data was non-normally distributed, medians with interquartile ranges (IQR) are presented. Associations between categorical variables were assessed using Pearson’s chi-squared test, or Fisher’s exact test where applicable. Means were compared across arms using the Student’s t-test and across timepoints using the paired Student’s t-test. Medians were compared across arms using the Wilcoxon rank-sum test and across timepoints using the Wilcoxon matched-pairs signed-rank test. Median serum estradiol was compared across arms and across timepoints using a mixed-effects linear regression with random effects for site and participant. Risk/Rate ratios (RR) for differences between arms and timepoints were estimated using generalised linear models with site and participant as a random effects and robust standard errors. Spearman correlation coefficients were calculated between serum estradiol and clinical and behavioral outcomes. Results were considered significant for p < 0.05.

**Ethics approval and consent**

Ethical approval was obtained from the Faculty of Health Sciences Human Research Ethics Committee (FHS HREC, M180528) of the University of Witwatersrand, and from the East London Hospital Institutional Ethics Committee. Permission to conduct the study was obtained from the Provincial Departments of Health of Eastern Cape and KwaZulu-Natal. Written informed consent was obtained from all women to participate in the WHICH study. Research staff with Good Clinical Practice certification and specific training in the recruitment procedures conducted recruitment. Informed consent complied with requirements for research on human subjects. Both sites had active Community Advisory Boards who approved the study at the planning stage.

**References**

1. Harris PA, Taylor R, Minor BL, Elliott V, Fernandez M, O'Neal L, et al. The REDCap consortium: Building an international community of software platform partners. J Biomed Inform. 2019; 95:103208.

2. Beck AT, Ward CH, Mendelson M, Mock J, Erbaugh J. An inventory for measuring depression. Arch Gen Psychiatry. 1961; 4:561-71.

3. Feldblum PJ, Caraway J, Bahamondes L, El-Shafei M, Quan Ha D, Morales E, et al. Randomized assignment to copper IUD or depot-medroxyprogesterone acetate: feasibility of enrollment, continuation and disease ascertainment. Contraception. 2005; 72(3):187-91.

4. Lawrie TA, Hofmeyr GJ, De Jager M, Berk M, Paiker J, Viljoen E. A double-blind randomised placebo controlled trial of postnatal norethisterone enanthate: the effect on postnatal depression and serum hormones. Br J Obstet Gynaecol. 1998; 105(10):1082-90.
