# Supplementary material for: Misreporting contraceptive use and the association of peak study progestin levels with weight and BMI among women randomized to the progestin-only injectable contraceptives DMPA-IM and NET-EN
Source: PLoS One. 2023 Dec 22;18(12):e0295959. doi: 10.1371/journal.pone.0295959 (PMC10745193; doi:10.1371/journal.pone.0295959)
Supplement: S2 Protocol — (DOCX) [file pone.0295959.s012.docx]

**S2 Protocol**

**UHPLC-MS/MS Progestin quantification methods**

**Progestin measurements**

Progestins were measured by UHPLC-MS/MS on stored samples from 435 WHICH study participants at D0 and 25W.

**Solvents and steroids**

Methyl tert-butyl ether (MTBE), LC-MS grade water, LC-MS grade methanol, LC-MS grade formic acid, ammonium fluoride (NH_4_F) and non-stripped human serum were purchased from Sigma-Aldrich (South Africa). MPA, NET, ETG, NES and GES were also purchased from Sigma-Aldrich (South Africa). LNG was purchased from the United States Pharmacopoeia (USP, Rockville, MD, USA), while the internal standards medroxyprogesterone-6,6,6-d3 17-acetate-2,2,2-d3 (MPA-d6) and norethindrone-2,2,4,6,6,10-d6 (NET-d6) were purchased from Toronto Research Chemicals (North York, ON, Canada). Individual stock solutions of the six progestins (1 mg/mL), as well as an internal standard stock solution (containing 0.5 ng/mL each of MPA-d6 and NET-d6), were prepared in ethanol and stored at -20°C until use. Human serum was heat inactivated by incubating at 56°C for 30 mins. Thereafter the heat-inactivated serum was stripped twice by incubating in activated charcoal (2.5% (w/v) charcoal, 0.25% (w/v) dextran, 0.1 M Tris-HCL pH 8) for 30 mins at 45°C, centrifuged at 5000 x g for 5 mins to pellet the charcoal and passed through a 0.22 μm filter.

**Calibration curve and internal quality control (IQC) sample preparation**

The individual progestin stock solutions were used to prepare two standard master mixes (1 µg/mL and 1 ng/mL) containing all the above-mentioned progestins in ethanol. These standard master mixes were subsequently used to prepare a twelve-point calibration curve, which included calibrations at 0, 0.01, 0.025, 0.05, 0.1, 0.25, 0.5, 1, 5, 10, 25 and 50 ng/mL, by addition of the appropriate volume of the standard master mix to stripped human serum (500 μL final volume). Three independent calibration series were prepared, extracted and quantified with each batch of serum samples. IQC samples were prepared in bulk (10 mL) by spiking stripped human serum with the progestin master mixes at four concentrations spanning the expected D0 and 25W progestin concentrations (0.05, 0.5, 5 and 50 ng/mL final concentration). Thereafter, each of the four homogeneous IQC’s were aliquoted (500 μL) and stored at -20°C until use. An aliquot of each IQC was thawed, extracted and quantified with each batch of serum samples.

**Progestin extraction**

Progestins were extracted by mixing 500 μL calibration curve standard, IQC or serum samples with 100 mL ultra-pure water containing 5 ng MPA-d6 and 5 ng NET-d6 (10 μL of 0.5 ng/μL stock) in clean disposable glass tubes. Thereafter the samples were loaded onto supported liquid extraction Isolute (SLE+) columns (Biotage, Uppsala, Sweden) and incubated for 5 minutes. The progestins were eluted into clean disposable glass tubes with 3 × 2 mL MTBE and subsequently dried under a stream of nitrogen gas at 40°C. The glass test tubes containing the extracted progestins were rinsed with 2 mL MTBE and dried again. The progestins were then carefully resuspended in 100 μL 1:1 methanol:water (v/v) and stored at -20°C prior to analysis.

**Instrument and chromatographic conditions for UHPLC-MS/MS**

The progestins were separated using an ACQUITY UPLC system (Waters Corporation, Milford, USA) and an ACQUITY UPLC HSS T3 column (2.1 mm x 50 mm, 1.8 μm particle size) (Waters Corporation, Milford, USA), coupled to an ACQUITY UPLC HSS T3 VanGuard, (pre-column (2.1 mm X 5 mm, 1.8 µm) (Waters Corporation, Milford, USA), at the Central Analytical Facility at Stellenbosch University. The column temperature was 60°C and the injection volume was 10 μL. The mobile phase consisted of 0.1% (v/v) formic acid in water (Mobile phase A) and methanol (Mobile phase B (MPB)). A gradient inlet method was used to separate the steroids using a constant flow rate of 0.60 mL/min according to the following protocol: 45% MPB from 0-5 min; 75% MPB from 5-5.10 min; 100% MPB from 5.10-6.50 min; 45% MPB from 6.50-7.50 min and hold for re-equilibration.

Post column infusion of NH_4_F [1] (3 mM in 50% methanol) at a constant flow rate of 20.0 mL/min using the fluidics system on the mass spectrometer under full software control was performed to enhance the ionisation of the progestins. Quantitative mass spectrometric detection was carried out using a Xevo TQ-S triple quadrupole mass spectrometer (Waters, Milford, USA). All progestins were analyzed in multiple reaction monitoring (MRM) mode using an electrospray probe in the positive ionisation mode (ESI+). The following settings were used: capillary voltage of 3.7 kV, desolvation temperature 350°C, desolvation gas 650 L/h and cone gas 150 L/h. MRM transitions and retention times are included in S1 Table.

**Extraction efficiency and matrix effects**

We determined the recovery of each steroid (extraction efficiency) by spiking six separate aliquots of stripped serum with the progestin master mix (5 ng/mL final) and internal standard mix (5 ng of each MPA-d6 and NET-d6), both before (pre) and after extraction, but before the dry-down step (post). Matrix effects were determined by spiking six individual vials of MTBE with progestin master mix (5 ng/mL final) and internal standard mix (5 ng each of MPA-d6 and 5 ng NET-d6) before drying down (no). Recovery and matrix effects were then determined as follows:

Recovery (%) = [Concentration (pre)/Concentration (post)] x 100

Matrix effects (%) = [(Concentration (post) – Concentration (no)) / Concentration (no)] x 100

Mean values between 80 - 120% for recovery and between -20 – 20% for matrix effects were considered acceptable (S2 Table).

**Accuracy and precision**

Accuracy was determined by individually spiking 10 aliquots of stripped human serum with progestin master mix at four different concentrations (0.05, 0.1, 0.5 and 10 ng/mL final). Thereafter internal standard mix was added, and samples extracted as above. A bias of the observed concentration and the nominal concentration between -20% and +20% was considered acceptable (S2 Table).

Precision was determined by bulk-preparing stripped human serum spiked with progestin master mix at four different concentrations (0.05, 0.1, 0.5 and 10 ng/mL final) and internal standard mix. These homogeneous samples were then aliquoted (500 µL) and each aliquot (n=10) was extracted and quantified in the same batch to assess intra-assay precision. Inter-assay precision was determined by quantifying the IQC samples in parallel with each batch of serum samples analyzed over the course of ten independent UHPLC-MS/MS runs. A coefficient of variation (CV) ≤ 20% was considered acceptable (S2 Table).

**Limits of detection and quantification**

The limit of detection (LOD) for each progestin was defined as the lowest concentration at which the signal to-noise (S/N) ratio of the quantifier ion was > three. The lower limit of quantification (LLOQ) for each progestin was defined as the lowest concentration for each progestin at which: the S/N ratio of the quantifier ion was > three; the S/N ration of the qualifier ion was > 10; with an acceptable precision [CV ≤ 20%] and a bias not > ±20%. The upper limit of quantification (ULOQ) was defined as the maximum concentration on the linear region of the calibration curve with an acceptable precision [CV ≤ 20%] and a bias not > ±20% (S3 Table).

**UHPLC-MS/MS method validation**

Acceptable levels of recovery (% extraction efficiency) and matrix effects were obtained, and accuracy (% BIAS) and precision (% CV) were both less than 15% (S2 Table). Linear calibration curves were obtained for each progestin for concentration ranging from their LLOQ to the ULOQ (R^2^>0.99).

**References**

1. Schiffer L, Shaheen F, Gilligan LC, Storbeck KH, Hawley JM, Keevil BG, et al. Multi-steroid profiling by UHPLC-MS/MS with post-column infusion of ammonium fluoride. J Chromatogr B Analyt Technol Biomed Life Sci. 2022; 1209:123413.
